# Expression of enterotoxin-coding genes in methicillin-resistant *Staphylococcus aureus* strains isolated from Mexican haemodialysis patients

**DOI:** 10.1186/s12941-014-0055-z

**Published:** 2014-11-25

**Authors:** Gloria Luz Paniagua-Contreras, Eric Monroy-Pérez, Felipe Vaca-Paniagua, José Raymundo Rodríguez-Moctezuma, Sergio Vaca

**Affiliations:** FES-Iztacala, Universidad Nacional Autónoma de Mexico, Av de Los Barrios 1, Los Reyes Iztacala, Tlalnepantla 54090 Edo de México, México; Instituto Mexicano del Seguro Social, G. Baz esq. F. Gómez, Tlalnepantla Edo de México, México

**Keywords:** MRSA, Haemodialysis catheter, Enterotoxins

## Abstract

**Background:**

Methicillin-resistant *Staphylococcus aureus* (MRSA) causes severe catheter-related infections in haemodialysis patients ranging from local-site infections and septic thrombophlebitis to bacteraemia but the associated virulence factors and exotoxins remain unclear.

**Findings:**

We employed an *in vitro* infection model using reconstituted human epithelium (RHE) to analyse the expression profiles of 4 virulence genes and 12 exotoxin-coding virulence genes in 21 MRSA strains isolated from catheter-related infections in 21 Mexican patients undergoing haemodialysis.

All 21 strains (100%) expressed the *seg, seh, sei, eta, etb,* or *hla* genes coding staphylococcal toxins. Eleven MRSA strains (52.3%) expressed the *sea* gene coding staphylococcal enterotoxin A, and two strains (9.5%) expressed the *v8* gene coding serine protease. The *tst*, *chp,* and *arcA* genes coding toxic shock syndrome toxin 1, chemotaxis inhibitory protein, and arginine deiminase, respectively, were expressed in separate single strains (4.7%). The most frequent expression profile (42.8% of the strains) comprised *seg, seh, sei, eta, etb,* and *hla*.

**Conclusion:**

It is likely that the SEG, SEH, SEI, ETA, ETB, and Hla toxins may play a role in MRSA catheter-related infections. Consideration of these toxins in the development of a vaccine or as targets for monoclonal antibody therapy could provide an improved therapeutic strategy for the treatment of catheter-related infections in haemodialysis patients.

## Findings

Methicillin-resistant *Staphylococcus aureus* (MRSA) is known to produce catheter-associated bacteraemia in haemodialysis patients because of its ability to form a biofilm in the interior of endovascular catheters [1]. *S. aureus* is able to produce several exotoxins with superantigen activity, including staphylococcal enterotoxins (SEA to SEE and SEG to SEJ) encoded by the genes *sea, seb, sec, sed, see, seg, seh, sei,* and *sej*; toxic shock syndrome toxin 1 (TSST-1, encoded by *tst*) and exfoliative toxins (ETA and ETB, encoded by *eta* and *etb*) [2]. Other virulence factors in *S. aureus* are encoded by the genes *hla* (alpha toxin), *chp* (chemotaxis inhibitory protein), *arcA* (arginine deiminase), and *v8* (serine protease V8) [3]. The expression of these factors is tightly regulated during growth, and the accessory gene regulator (*agr*) system, also known as the quorum-sensing system, plays a central role in the regulation of virulence factors [4]. The frequency of MRSA infections has increased, and nosocomial infections are now a serious problem because of the limited number of effective antibiotics available for treatment [5].

We recently analysed the expression profiles of genes encoding adhesins in MRSA strains isolated from catheter-related infections in patients undergoing haemodialysis [6]; however, it is not known whether these strains carry and express genes encoding enterotoxins. In this study, we employed an *in vitro* infection model using reconstituted human epithelium (RHE) to analyse the expression profiles of genes encoding pyrogenic exotoxins in our collection of MRSA strains isolated from catheter-related infections in Mexican patients undergoing haemodialysis.

The 21 MRSA strains analysed in this study were isolated from catheter-related infections in 21 Mexican patients and have been described previously [6]. Bacterial DNA was extracted using the Wizard Genomic DNA Purification Kit (Promega, Fitchburg, WI, USA).

Reconstituted human epithelium (RHE; SkinEthic Laboratories, Nice, France) consists of human epithelial cells cultured on polycarbonate filters *in vitro* at the air-liquid interface in serum-free conditions in a defined medium based on the MCDB-153 medium (Clonetics, San Diego, CA, USA) containing 5 μg insulin/mL. In total, 2 × 10^6^ *S. aureus* cells suspended in 50 μL of 0.1 M phosphate-buffered saline (PBS) were inoculated onto the surface of the RHE and incubated at 37°C for 72 h under 5% CO_2_ and saturated humidity. The media were changed every 24 h.

*S. aureus* RNA purification and reverse transcription were performed as previously described [6]. The primers for real-time PCR were described previously for the following genes *sea, seb, sec, sed, see, seg, seh, sei, sej, tst, eta*, *etb, hla, chp, arcA,* and *v8* [3]. The Rotor-Gene SYBR Green PCR kit (Qiagen, Hilden, Germany) was used for real-time PCR expression profiling according with the manufacturer’s instructions. *gyrB* (DNA gyrase B) was used as a reference gene. *S. epidermidis* ATCC 35984 and *Escherichia coli* ATCC 11775 were used as negative controls. *S. aureus* ATCC 33592 was used as the positive control.

All MRSA strains (n = 21) expressed 6 genes, that is, *seg, seh, sei, eta, etb, and hla,* of the 16 genes studied during *in vitro* infection of RHE. *tst, chp*, and *arcA* were expressed in separate single strains (4.7%), and *v8* was expressed by 2 strains (9.5%; Table 1). Six distinct expression profiles of virulence markers were found during MRSA infection of RHE. Profile 1, composed of 6 genes (*seg, seh, sei, eta, etb,* and *hla*), was seen for nine (42.3%) MRSA isolates, and profile 2, composed of 7 genes (*sea, seg, seh, sei, eta, etb,* and *hla*), was seen for seven strains (33.3%). Profile 3, formed by 8 genes (*sea, seg, seh, sei, eta, etb, hla,* and *v8*), was found in 2 (9.5%) MRSA isolates. Three distinct profiles comprising 7–8 expressed genes in only one MRSA strain each were also found.

**Table 1:** **Expression of pyrogenic exotoxins genes of the MRSA strains**

	**STRAINS**	**S-10**	**S-16**	**S-19**	**S-22**	**S-36**	**S-52**	**S-58**	**S-59**	**S-66**	**S-73**	**S-75**	**S-76**	**S-77**	**S-79**	**S-82**	**S-93**	**S-103**	**S-106**	**S-107**	**S-8**	**S-108**	**No. (%)**
**Pyrogenic exotoxins expression**	***sea***	+	-	+	-	-	-	-	-	-	+	+	+	+	+	+	-	+	+	-	-	+	11 (52.3)
	***seb***	-	-	-	-	-	-	-	-	-	-	-	-	-	-	-	-	-	-	-	-	-	0 (0)
	***sec***	-	-	-	-	-	-	-	-	-	-	-	-	-	-	-	-	-	-	-	-	-	0 (0)
	***sed***	-	-	-	-	-	-	-	-	-	-	-	-	-	-	-	-	-	-	-	-	-	0 (0)
	***see***	-	-	-	-	-	-	-	-	-	-	-	-	-	-	-	-	-	-	-	-	-	0 (0)
	***seg***	+	+	+	+	+	+	+	+	+	+	+	+	+	+	+	+	+	+	+	+	+	21 (100)
	***seh***	+	+	+	+	+	+	+	+	+	+	+	+	+	+	+	+	+	+	+	+	+	21 (100)
	***sei***	+	+	+	+	+	+	+	+	+	+	+	+	+	+	+	+	+	+	+	+	+	21 (100)
	***sej***	-	-	-	-	-	-	-	-	-	-	-	-	-	-	-	-	-	-	-	-	-	0 (0)
	***tst***	-	+	-	-	-	-	-	-	-	-	-	-	-	-	-	-	-	-	-	-	-	1 (4.7)
	***eta***	+	+	+	+	+	+	+	+	+	+	+	+	+	+	+	+	+	+	+	+	+	21 (100)
	***etb***	+	+	+	+	+	+	+	+	+	+	+	+	+	+	+	+	+	+	+	+	+	21 (100)
**Others genes expression**	***hla***	+	+	+	+	+	+	+	+	+	+	+	+	+	+	+	+	+	+	+	+	+	21 (100)
	***chp***	-	-	-	-	-	-	-	-	-	-	-	-	-	-	+	-	-	-	-	-	-	1 (4.7)
	***arcA***	-	-	-	-	-	-	-	-	-	-	-	-	-	+	-	-	-	-	-	-	-	1 (4.7)
	***v8***	-	-	-	-	-	-	-	-	-	-	+	+	-	-	-	-	-	-	-	-	-	2 (9.5)

MRSA strains previously reported as belonging to the *spa* type t895 and having identical pulsed-field gel electrophoresis (PFGE) profiles (S-22 and S36; S59 and S-66) [6] showed identical expression profiles of pyrogenic exotoxin genes. Additionally, the toxin gene expression profiles of MRSA strains S-77, S-79 and S-82, which have a distinct PFGE profile but the same *spa* type (t895) [6], differed by one gene: *arcA* (S-79) or *chp* (S-82).

### Expression of enterotoxin-coding genes in MRSA

Catheter-related bacteraemia caused by MRSA in patients who have end-stage renal disease and are undergoing chronic haemodialysis is a serious health problem [1]. Several studies have characterised *S. aureus* virulence factors in animal models [7-9]. In this study, we employed an *in vitro* infection model using RHE to analyse the expression profiles of genes encoding exotoxins in MRSA strains isolated from catheter-related infections in Mexican patients undergoing haemodialysis. All of the MRSA strains studied here expressed an important set of virulence factors. All of the strains expressed three staphylococcal enterotoxins (SEG, SEH, and SEI), two exfoliative toxins (ETA and ETB), and the alpha toxin (Hla) (Table 1). We had previously showed that these strains also expressed the global regulator of multiple virulence factors, *agr* (18/21 strains expressed agr I, 3/21 expressed agr II) [6], which had previously been associated with suppurative infections [10]. We also showed that 12 of 21 of these strains harboured SCC*mec* type IV, 6 of 21 harboured SCC*mec* type II, and 3 of 21 harboured SCC*mec* type I [6].

The frequency of genes encoding toxins has been previously described for *S. aureus* strains related to different types of infections [3,11]; however, to our knowledge, this is the first time that the expression of genes encoding pyrogenic toxins in MRSA strains associated with catheter-related infections was profiled.

The results presented here show that it is likely that the SEG, SEH, SEI, ETA, ETB, and Hla toxins may play a role in the pathogenesis of MRSA catheter-related infections. Comparative quantification of the expression of these genes in these MRSA and in other *S. aureus* isolates could further support this point. Considering these toxins in the development of a vaccine, or as targets for monoclonal antibody therapy, could provide an improved therapeutic strategy for the prevention or treatment of catheter-related infections.

## Availability of supporting data

The data supporting the results of this study are included within this article.
